# Treatment Interventions for Women With Alcohol Use Disorder

**DOI:** 10.35946/arcr.v40.2.08

**Published:** 2020-07-30

**Authors:** Barbara S. McCrady, Elizabeth E. Epstein, Kathryn F. Fokas

**Affiliations:** 1 Center on Alcoholism, Substance Abuse, and Addictions, University of New Mexico, Albuquerque, New Mexico; 2 Department of Psychiatry, University of Massachusetts Medical School, Worcester, Massachusetts; 3 Department of Psychology, University of New Mexico, Albuquerque, New Mexico

**Keywords:** alcohol use disorder, barriers, mechanisms of change, outcomes, treatment, women

## Abstract

Women with alcohol use disorder (AUD) experience more barriers to AUD treatment and are less likely to access treatment than men with AUD. A literature review identified several barriers to women seeking help: low perception of a need for treatment; guilt and shame; co-occurring disorders; employment, economic, and health insurance disparities; childcare responsibilities; and fear of child protective services. Women entering treatment present with more severe AUD and more complex psychological, social, and service needs than men. Treatment program elements that may reduce barriers to AUD treatment include provision of childcare, prenatal care, treatment for co-occurring psychological problems, and supplemental social services. Research has suggested that outcomes for women are best when treatment is provided in women-only programs that include female-specific content. To date, research on treatments tailored to the individual needs of women is limited, but research on mechanisms of change has suggested the importance of targeting anxiety and depression, affiliative statements in treatment, abstinence self-efficacy, coping skills, autonomy, and social support for abstinence. Future research should focus on early interventions, linkages between primary care or mental health clinics and AUD treatment settings, and integrated treatments for co-occurring AUD and other disorders. Further research should also explore novel treatment delivery approaches such as digital platforms and peer support groups.

## INTRODUCTION

Historically, women with alcohol use disorder (AUD) have been an underserved population. In the United States, more than 5 million adult women, or 4.2% of the adult female population, meet criteria for current AUD.^1^ Although this percentage is half that of adult men (8.4%), among adolescents, more females than males meet criteria for current AUD (2.7% vs. 2.3%),^1^ and recent research has suggested that the gender gap in alcohol use and alcohol-related harm is narrowing.^2^ Heterogeneity in rates of AUD is found among different racial/ethnic groups, with higher rates among Black and Hispanic women than among White women,^3^ and rates of AUD among gender minority women also are higher than among heterosexual women.^4^

A smaller proportion of women than men received AUD treatment both in the past year^1^ (7.9% of adult women vs. 9.2% of adult men; 4.6% of adolescent females vs. 7.4% of adolescent males) and in their lifetime^5^ (15.0% of women and 22.0% of men with AUD who are younger than age 45). Utilization rates for treatment services by women and men do not differ across different racial/ethnic groups.^5^ Given the increasing rates of AUD among women and the lower rates of treatment utilization among women, a rethinking of AUD treatment for women is in order. The purpose of this article is to describe the barriers to treatment entry experienced by women with AUD, the unique characteristics and presenting concerns of women with AUD who do seek treatment, and the current knowledge about effective treatments. Sources of information for this review included a comprehensive review published in 2013,^6^ articles identified in a search in PsycINFO^®^ using the search terms “women,” “alcohol,” and “treatment,” and articles identified through selective reviews to identify key publications on trauma-informed treatment and substance use disorder (SUD) in female veterans.

## WOMEN SEEKING AUD TREATMENT

Women seeking AUD treatment differ from men in their sociodemographic characteristics and psychological profiles. They experience some unique barriers to accessing treatment and present to treatment with some needs that differ from men in AUD treatment.

### Characteristics of Women With AUD at Treatment Entry

Women seeking AUD treatment vary along a number of dimensions that may impact their access to treatment, treatment needs, and treatment response.

#### Sociodemographic characteristics and substance use

Women who present to AUD treatment often have markedly different characteristics and backgrounds than men in these treatment settings. Such distinctions among women include younger age, more severe alcohol and drug use histories, less education, lower income, higher unemployment, more housing needs, more children living at home, and higher parental stress.^6^ In terms of substance misuse, rates differ among subgroups. For example, non-Hispanic White and American Indian/Alaska Native women are more likely than women of other racial/ethnic groups to identify alcohol as their primary substance of use when entering treatment for SUD.^7^ Among pregnant women entering treatment for SUD, approximately 18% identified alcohol as their primary substance of use.^7^ In a study of women veterans with SUD, researchers found that entry into and engagement with treatment were associated with having a co-occurring psychological disorder and receiving services at facilities offering women’s treatment.^8^

#### Psychological co-occurrences

Compared to men, women who enter AUD/SUD treatment generally report higher levels of physical and mental health concerns. Rates of co-occurring disorders vary with the treatment setting and population. Epidemiologic data suggest that compared with men with AUD, women with AUD have a higher prevalence of co-occurring DSM-IV Axis I disorders (84.2% vs. 75.5%), a similar prevalence of other drug dependence (15.2% vs. 14.3%), a higher prevalence of mood and anxiety disorders (53.1% vs. 29.1% and 44.3% vs. 26.2%, respectively), and a similar prevalence of personality disorders (36.5% vs. 33.3%).^9^ A recent nationwide study of veterans with AUD found that women veterans had more psychological and substance use comorbidities than men.^10^ In addition, women in SUD treatment have a much higher prevalence (up to 80.0%) of lifetime physical, sexual, and/or emotional abuse and trauma, and concerns about current domestic violence are common.^11^ Rates of current post-traumatic stress disorder (PTSD) among women in SUD treatment range from 25.0% to 55.0%.^12^

### Barriers to Treatment

Women who do not receive AUD treatment have some sociodemographic difference from women in AUD treatment. For example, a sample of women with AUD who were not in treatment but perceived a need for treatment were less educated, had a family income less than $75,000, and were more likely to use psychotropic medications compared to those who did not perceive a need for treatment.^13^ Women experience both internal and external barriers to AUD treatment. These barriers may partially explain the gender discrepancy in treatment initiation rates and include low perception of need for treatment; guilt and shame stemming from the discrepancy between traditional gender expectations and societal views of women with AUD; depression and other co-occurring disorders; greater employment, economic, and health insurance disparities relative to men; childcare responsibilities; and fear of child protective services.^6^

Recent research has suggested that traditional gender expectations and lay beliefs about AUD may contribute to lower AUD treatment utilization among women. Lale and colleagues found that compared to men, women were more likely to attribute AUD to “bad character” and less likely to attribute AUD to genetics.^14^ Women also worry that they will be perceived as “bad mothers” and potentially lose custody of their children if they disclose having an alcohol problem.^7^ Relatedly, women are more likely than men to experience feelings of embarrassment, to experience fear, to have the belief that no one can help, and to have the belief that their problem is not serious enough to require AUD treatment.^15^ In addition to these intrapersonal barriers, women may experience less social support to enter AUD treatment than men do. Women with AUD are more likely than men to be in an intimate relationship with a partner who also has AUD,^16^ and women tend to have less spousal and family support for recovery.^17^ Further, women generally report more logistical barriers to treatment utilization, including greater difficulties with transportation, lack of available childcare, and inadequate insurance coverage.^17^

Compared to men, women are more likely to seek AUD treatment through a general versus substance use-specific health care sector^18^ or in the context of treatment at a general mental health clinical setting,^19^,^20^ and less likely to be court mandated to treatment.^21^ Women with AUD also generally report stressful life events and nonsubstance-related mental health concerns as their primary reasons for seeking treatment.^22^ Welfare, child welfare, and legal systems provide additional portals through which some women enter AUD treatment.^21^ Primary care physicians, gynecologists, and psychiatrists may benefit from focused training in identification and referral of women with AUD to offset the gender discrepancy observed in women’s entry into AUD treatment. Relatedly, women have shown a preference for AUD treatment settings that offer childcare.^23^ Thus, more easily accessible, children-friendly treatment centers with wide availability are also likely to improve treatment utilization among women with AUD.

## AUD TREATMENT SERVICES FOR WOMEN

### Treatment Retention

In general, the literature is mixed regarding AUD treatment attrition and gender differences.^6^ Previous studies have found that women tend to have longer inpatient stays and that longer inpatient stays are associated with an increase in sustained abstinence for women but not for men.^22^,^24^ Bravo and colleagues reported that women engaged in outpatient AUD treatment longer and discontinued treatment at a lower rate than men.^25^ In a comprehensive review, Greenfield and colleagues concluded that although there are no gender differences in attrition, predictors and mediators of treatment retention differ by gender.^23^ Predictors of better treatment retention among women include demographic variables, such as lower psychiatric impairment, higher socioeconomic status, and greater social support and stability,^23^ and program variables, such as female-specific treatment and facilities that allow children to stay with their mothers.^6^ A recent investigation of 1.8 million individuals who received SUD treatment at federally funded facilities found that, across treatment settings, women and men did not differ in rates of early discharge.^26^ However, when treatment settings were stratified by type (detoxification, residential, and ambulatory), women were more likely than men to leave detoxification treatment prematurely. The authors suggested that lower rates of female-specific services and higher rates of psychiatric co-occurring disorders within detoxification settings might have accounted for this gender difference.

### Treatment Outcome

In general, studies of mixed-gender treatment programs have found few gender differences in short-term outcomes for AUD across a range of interventions, samples, and sites, despite women at baseline generally presenting with more severe clinical issues.^6^ For example, in their analysis of five randomized clinical trials (RCTs) of intensive outpatient contingency management for AUD and SUD, Rash and Petry found no differences between men and women’s abstinence rates during the 3-month treatment period, although women initially presented with more financial, family/social, and psychiatric problems.^27^ Likewise, a study of a large outpatient AUD treatment cohort in Spain found no differences between men and women in alcohol consumption 1 year posttreatment, despite women presenting with more symptoms of dependence at baseline.^25^

Results have been more mixed regarding women’s long-term outcomes compared to men.^6^ In the same study from Spain described above, women had superior drinking outcomes compared to men at 5, 10, and 20 years posttreatment.^25^ Conversely, Litt and colleagues found that women had worse drinking outcomes than men in the 2 years following outpatient AUD treatment.^28^ These poorer outcomes may have been due to the nature of the active treatment, which focused on altering the participant’s social network to gain more support for abstinence; women in the study had less abstinence-supportive social networks and more difficulty altering these networks.

Historically, gender has typically not been taken into consideration in psychopharmacologic treatment for AUD, and women have been underrepresented in AUD medication trials.^29^ However, research has begun to improve in this area. A review by Agabio and colleagues found that too few studies of disulfiram had included women to test potential gender differences in response to this medication.^30^ There were a sufficient number of studies on acamprosate and naltrexone, which showed that both medications were generally efficacious for women; however, results of gender comparisons were too variable to draw firm conclusions. Canidate and colleagues conducted a systematic review of seven studies on naltrexone for the treatment of AUD among women.^31^ Among this limited number of studies, naltrexone was found to have a modest effect on drinking quantity and time of relapse but not on the overall frequency of drinking among women. The authors concluded that the effect of naltrexone on women is currently understudied. This Canidate article highlights the need to continue to use rigorous research designs to study differences in the efficacy of naltrexone on women versus men.

### Reducing Barriers to Treatment for Women

A comprehensive review identified six major elements of SUD treatment programs for women that reduce barriers to treatment and/or address women’s unique needs.^32^ These include the provision of childcare, prenatal care, women-only treatment, treatment for co-occurring mental health problems, a comprehensive approach to treatment, and supplemental services that address women-focused topics. Each of these elements was linked to favorable treatment outcomes. In a qualitative meta-synthesis of programs that included women and their children, several treatment processes were identified by different stakeholders (clients, clinicians, and program administrators) as instrumental to positive outcomes: developing a sense of agency, giving and receiving social support, engaging with program staff, fostering self-disclosure, recognizing self-destructive patterns of behavior, setting goals, and feeling motivated by the presence of children.^33^ Although some of these processes are common to any AUD treatment, it is necessary to recognize the unique blend of common and specific treatment processes that are effective for women in treatment with their children. Although studies have repeatedly identified the importance of including children-supportive services in women’s SUD treatment programs, a 2018 Substance Abuse and Mental Health Services Administration (SAMHSA) survey found that only 5.8% of SUD treatment facilities provided childcare and only 2.6% of residential programs provided beds for clients’ children.^34^

### Guiding Principles for Women’s AUD Treatment

Recognizing the unique treatment needs of women with AUD and SUD, SAMHSA published a set of evidence-based principles to guide gender-responsive treatment for women.^7^ These guidelines include several recommendations. For example, they recommend developing cultural competence to frame women’s AUD symptoms and treatment in their socioeconomics contexts (e.g., employment, income, housing). They suggest that providers acknowledge the unique significance of women’s relationships and attend to the “caregiver roles that women often assume throughout the course of their lives.” Relatedly, the guidelines address stigma by noting the importance of “recognizing that ascribed roles and gender expectations across cultures affect societal attitudes toward women who abuse substances.” Other recommendations state that SUD treatments for women adopt a trauma-informed approach, which often emphasizes women’s strengths, and address “women’s unique health concerns” through “an integrated and multidisciplinary approach.” The SAMHSA guidelines conclude that clinical treatment services (e.g., screening, mental health services), clinical support services (e.g., parenting education, job training), and community support services (e.g., childcare, transportation) would work collaboratively to facilitate comprehensive AUD treatment for women of diverse backgrounds.^7^

### Advances and Gaps in Treatment Development for Women

With increasing recognition of the unique clinical profiles of women with AUD has come increasing attention to whether AUD treatment programs are serving the needs of women. The 2018 SAMHSA annual survey of substance use treatment programs found that 49% of programs surveyed provided special programs or groups for women and 23% provided services for pregnant or postpartum women.^34^ In contrast, data from the Veterans Health Administration (VHA) revealed that most VHA facilities offered SUD services to women but that most of these services were generic rather than female-specific (85% vs. 30%).^35^

The need for specialized services for women has both an empirical and a clinical rationale. As reviewed earlier in this article, compared to men, women are less likely to seek AUD treatment, have different social contexts, present with different profiles of co-occurring disorders, and have a unique and complex set of service needs that may not be addressed in a standard, mixed-gender AUD treatment program.^9^,^36^ Thus, treatment programs and researchers have been seeking to create and evaluate services intended to attract women to AUD treatment and improve outcomes. AUD services for women vary along two dimensions—whether they are provided in a mixed-gender or women-only treatment setting and whether the content of the treatment is generic or tailored specifically to women’s clinical and other service needs.^37^ Thus, delivery of AUD treatment to women may occur in (a) mixed-gender programs with no female-specific programming, (b) mixed-gender programs with female-specific programming, (c) single-gender (women-only) programs with no female-specific programming, or (d) single-gender (women-only) programs with female-specific programming.

#### Mixed-gender versus single-gender treatment

Single-gender treatment services seem appealing because they have the potential to provide an environment in which women may feel more comfortable sharing emotional and personal information. For instance, it is possible that among women who have a history of trauma or abuse from men, single-gender treatment might be preferable because of the possibility that participation in a mixed-gender program could trigger trauma-related symptoms. In addition, given the broader literature on the relative interactional dominance of men in mixed-gender groups, women may have more opportunities to participate when in women-only groups.^38^ However, research on women’s treatment preferences yields a more nuanced picture. Although some research suggests that women prefer women-only groups,^23^ a narrative analysis of interviews with women with a range of SUD treatment experiences found that the women reported concerns and anxiety about being in women-only treatment because of their own history of dysfunctional relationships with women and their greater comfort in being with men.^39^ However, women in the study reported positive experiences once they entered women-only services.

Few studies have compared women’s outcomes from mixed-gender versus women-only programs that were not adapted with female-specific content. In one early study, Bride compared the outcomes for women who were in a mixed-gender program to the outcomes for women who later participated in the same program that had become a women-only program with no female-specific content.^40^ Outcomes were similar between the two samples.

More extensive research has compared mixed-gender to single-gender programs that incorporate female-specific themes, services, or content. For example, interviewed providers of services for female veterans with SUD identified five female-specific themes and services that they viewed as key to treatment: a focus on safety; scheduling that accommodates women’s work and family responsibilities; flexibility in the resources provided; staff trained in serving women’s clinical needs; provision of on-site childcare; and a positive, supportive, nonconfrontational treatment environment.^41^ Although some of these treatment elements may be relevant to treatment for any patient with SUD, the combination of these elements was seen as key to successful treatment for the female veteran population. In addition to treatment elements, female-specific content has focused on clinical issues of particular significance to women, such as trauma, physical abuse, relationships, parenting, assertiveness, and treatment of co-occurring disorders.

One of the earliest studies of women-only treatment with female-specific content was the Early Treatment of Women with Alcohol Addiction (EWA) study.^42^ A 2-year follow-up of women found better clinical outcomes in the EWA than mixed-gender treatment, and a long-term study of mortality revealed lower mortality rates for younger women who participated in the EWA program than the mixed-gender treatment.^43^ A later study of a large sample of women in women-only versus mixed-gender residential SUD treatment found that women were twice as likely to complete the women-only treatment and that higher retention was associated with higher rates of abstinence posttreatment.^44^,^45^ More recent studies have found that (a) treatment retention and entry to aftercare were enhanced by gender-specific services in an intensive treatment program that also provided transitional housing, particularly for women who completed residential treatment;^46^ (b) women-only treatment predicted better legal and drug outcomes but no differences in alcohol use outcomes;^47^ and (c) women in the single-gender treatment had significantly less substance use (participants were primary stimulant users) and less criminal activity than those in the mixed-gender treatment.^48^ In contrast, Kaskutas and colleagues found that a mixed-gender, comprehensive, hospital-based treatment resulted in better alcohol abstinence outcomes than women-only treatment and was superior to generic, community-based, mixed-gender treatment.^49^

#### Single-gender treatment with no female-specific programming

Some empirically supported treatments have been tested in female samples with any adaptation of the treatment to women’s treatment needs. Two studies compared behavioral couple therapy to individual treatment for women with AUD and their male partners.^50^,^51^ O’Farrell and colleagues compared behavioral couple therapy to individual treatment for women with SUD and their male partners.^52^ All three studies found that the behavioral couple therapy led to positive changes in alcohol or drug use, with better alcohol or drug use outcomes for the women receiving couple therapy. In their study, McCrady and colleagues found that women presenting with higher levels of relationship distress and women with co-occurring Axis I or II disorders had greater improvements in drinking.^50^ Note, however, that couple therapy is a modality available to only a small proportion of the population of women with AUD. Notably, when given the choice, even women with male partners indicated a preference for individual rather than couple therapy, stating that they wanted to work on their own problems, did not see their partners as supportive, or thought the logistics of scheduling couple sessions was too difficult.^53^

Chronic care models for persons with serious mental illness and SUD are another empirically supported approach that has been tested in female samples without female-specific programming. These models have been developed and tested with homeless women who have AUD. The chronic care model emphasizes availability of a primary care provider, care management, education about alcohol, and referral to addiction services. Compared to women who received treatment as usual in a health care clinic for homeless women, women who participated in the chronic care program engaged with more SUD treatment services in the 3 months after starting the program.^54^

#### Single-gender treatment with female-specific programming

There has been substantial research on women-only treatment with female-specific content. For example, Polcin and colleagues compared intensive, nine-session motivational interviewing (MI) for women with standard one-session MI.^55^ For the intensive treatment, therapists were trained to use MI to focus on alcohol use as well as female-specific themes—such as personal relationships, issues related to parenting, abuse, and barriers to treatment—and other psychological concerns, such as low self-esteem or co-occurring disorders. Compliance with the treatment was high (80% of heavy drinkers completed at least seven sessions), and women receiving intensive MI reduced their drinking more than women receiving standard MI. Connors and Walitzer developed and tested an intervention to help heavy-drinking, nonalcohol-dependent women reduce their drinking.^56^,^57^ The intervention focused on skills to reduce drinking and other life skills believed to be relevant to women, such as problem-solving, communication and assertiveness, and strategies to enhance their social support system. Compared to treatment focused only on drinking, women who also received the life skills interventions and booster sessions had outcomes that were more positive.

Another single-gender treatment with women-specific programming was developed by Epstein and colleagues. The outpatient, female-specific cognitive behavioral treatment (FS-CBT) was an adaptation of a the gender-neutral cognitive behavior therapy manual-guided treatment for AUD.^58^ The FS-CBT manual (a) highlighted two clinical themes meaningful to women, self-care and autonomy; (b) included female-specific interventions focused on coping with negative emotions and developing/enhancing women’s social network supportive of abstinence; and (c) provided women-specific examples throughout to personalize the material to each woman’s issues, such as dealing with heavy drinkers in the social network, parenting, life-stage transitions, trauma, self-esteem, and relationships.^59^ In an RCT comparing FS-CBT to an evidence-based, gender-neutral CBT for AUD, Epstein and McCrady found that women in both treatment conditions were highly engaged, reported a high level of satisfaction with the treatment, significantly reduced their drinking, and improved in other areas of life functioning such as depression, anxiety, autonomy, and sociotropy.^58^ There were no treatment condition effects, and the FS-CBT treatment was equally effective as the gender-neutral one. In a subsequent RCT, Epstein and colleagues tested the individual modality FS-CBT treatment versus a new group therapy format of the same contents in a “pure comparison” design.^60^ Both FS-CBT treatment modalities (individual and group therapy) resulted in significant positive changes in drinking, depression, anxiety, coping skills, self-confidence, interpersonal functioning, and self-care even though treatment attendance and therapeutic alliance were greater in the individual FS-CBT condition. Cost-effectiveness analyses favored the group format.^61^

In a pilot study, Greenfield and colleagues tested a women-only Women’s Recovery Group (WRG, *n* = 16) for SUD against mixed-gender Group Drug Counseling (GDC, *n* = 7 women, 10 men).^62^ WRG included cognitive behavioral and relapse prevention elements, as well as “repair work” relevant for women (repairing SUD-related damage to relationships and self, and learning to enjoy life without substances).^63^ GDC was a traditional mixed-gender treatment program focused on substance-related topics with no gender-specific content. During treatment, the groups did not differ in substance^62^ or psychiatric improvement;^64^ however, women in WRG continued to reduce substance use in the 6 months posttreatment, and also reported higher satisfaction with the treatment they received.

In a subsequent, larger RCT,^65^ with a similar design except that the WRG groups offered rolling admission, outcomes of 52 women in WRG were compared with those of 48 women in GDC (with 58 men in GDC). All participants had SUD or AUD. Women in both treatments reduced drinking, and there were no treatment condition differences in within- or posttreatment drinking outcomes. Because WRG had both a women-only group composition and female-specific content compared to GDC, which had both a mixed-gender format and no female-specific content, it is unclear whether study results were linked to group composition, female-specific content, or both, but both the pilot and the larger RCT demonstrated that WRG is at least comparable to a typical “treatment-as-usual” such as a mixed-gender GDC in community settings. The authors also noted that the WRG in the larger trial was delivered on a rolling admissions basis and suggested that the revised format may have diluted the impact of the WRG.

In a series of three studies on putative mechanisms of change in WRG, secondary analyses of the pilot and/or larger RCT data from studies just described here above, showed that more affiliative statements were made in WRG than GDC^66^,^67^ and that more affiliative statements were associated positively with women’s drinking outcomes during and 6 months after treatment, particularly in the WRG condition.^68^ Sugarman and colleagues created and piloted (for feasibility, acceptability, and satisfaction) a web-based, gender-specific individual psychoeducation intervention based on WRG content.^69^ The gender-specific modules might ultimately comprise a female-specific component of care to be delivered in a mixed-gender setting.

Najavits and colleagues reported an RCT comparing the A Woman’s Path to Recovery (WPR) model to the gender-neutral 12-Step Facilitation (TSF) model for women veterans with SUD, the majority of whom (i.e., more than 74%) had current AUD.^70^ The WPR model is based on cognitive behavioral, interpersonal, and emotive therapy methods, and theory on gender differences in addiction and recovery. The “exploration” phase of the treatment highlights five themes: “body and sexuality, stress, relationships, trauma and violence, and thrill-seeking.”^70^^(p211)^ The “healing” section covers “recovery methods in four domains—relationships, beliefs, actions, and feelings.”^70^^(p211)^ Both WPR and TSF were single-gender groups, facilitated by women clinicians, and provided compensation to offset potential childcare costs or other financial barriers to participation. The treatments resulted in similar improvements in alcohol and drug use, coping skills, and psychiatric functioning. The authors noted that female-specific treatment content might be less relevant to veterans than to their civilian counterparts because male-dominated military culture may diminish traditional gender experiences for women.

In summary, several forms of empirically supported treatments have been tested and found to be efficacious with women, and several women-only treatments with female-specific content have been tested in rigorous RCTs. Overall, most of these studies have found limited evidence for superior alcohol use outcomes, but several of these studies have found greater satisfaction with the female-specific format and treatment content. Because these programs are appealing to women, they may increase women’s utilization of AUD treatment, and enhance both engagement and retention in AUD treatment.

### Treatment for Co-occurring Disorders

Treatment for co-occurring disorders may be indicated for the many women with AUD who present with additional mental health concerns. Interventions that address the co-occurrence of AUD with trauma and PTSD, mood disorders, and borderline personality disorder may be especially relevant for women.

#### Trauma

Given the highly elevated rates of trauma among women with AUD/SUD, SAMHSA has suggested that treatment for this population may benefit from adopting principles of trauma-informed care.^7^ A trauma-informed approach recognizes the prevalence and impact of trauma in women with AUD and adjusts treatment accordingly, even if clients do not meet diagnostic criteria for PTSD. Trauma-informed AUD treatment does not need to target trauma explicitly, but rather may consider trauma in the assessment and planning phases of treatment. For example, SAMHSA recommends that AUD treatment providers should assess women at intake for trauma histories and PTSD symptomatology and refer clients with severe symptomatology to providers who have experience working with traumatized populations (i.e., if they lack such experience themselves). Another recommendation is to “avoid triggering trauma reactions or re-traumatizing women.” For example, violating a client’s trust or disregarding a client’s emotions or experiences may trigger trauma reactions. SAMHSA also recommends that programs should “adjust staff behavior” and modify the treatment environment “to support clients’ coping capacities and safety concerns.” Specific strategies may include ensuring that urine specimens are collected in a private setting and establishing consistency in the treatment program’s routines and enforcement of rules. In addition, AUD treatment providers should “allow survivors to manage their trauma symptoms” in a manner conducive to AUD treatment engagement and success. For example, allowing clients to express strong feelings without facing judgment and explicitly addressing trauma only when a client is ready are considered trauma-informed approaches. Finally, SAMHSA recommends that trauma-informed AUD treatment for women should “emphasize skills and strengths, interactive education, growth, and change beyond stabilization.” Specific skills to incorporate into treatment may include assertiveness training and relaxation techniques.

Covington developed the Helping Women Recover program for the treatment of SUD.^71^ Following the principles of trauma-informed care, this treatment aims to provide a “healing” (i.e., safe, empowering, relational) environment that emphasizes strengths and is sensitive to cultural and gender issues. Treatment modules include topics hypothesized to be essential to women’s recovery: a focus on self and the integration of roles with feelings, thoughts, and attitudes; healthy interpersonal relationships; sexuality; and spirituality. Covington also developed the Beyond Trauma: A Healing Journey for Women treatment program, which teaches women how to identify trauma and other forms of abuse, helps them understand typical reactions to trauma and abuse, and fosters the development of coping skills.^72^ In an RCT with incarcerated women, 77% of whom were primary stimulant users, Messina and colleagues integrated the Helping Women Recover and Beyond Trauma protocols into a gender-responsive treatment (GRT) program.^73^ GRT was compared to a standard prison-based therapeutic community (TC), which, like GRT, was single-gender and targeted SUD, but unlike GRT did not focus on gender-specific issues or trauma histories. Both conditions improved women’s psychological well-being and alcohol use outcomes, but women in GRT also had more favorable outcomes for drug use, length of aftercare treatment engagement, and rate of reincarceration in the year following release from parole. A subsequent analysis showed that women with physical/sexual abuse histories had significantly better posttreatment depression and substance use outcomes following GRT than TC.^74^

An extension of trauma-informed care is treatment for co-occurring SUD and PTSD. In general, this co-occurrence is complex and difficult to treat because SUD and PTSD are reciprocally functional and often exacerbate each other.^75^,^76^ Drinking or drug use often functions to self-medicate PTSD symptoms and enable avoidance of remembering traumatic events. Reducing substance use may initially intensify PTSD symptoms and thus predispose the client to relapse. An increasing focus has emerged on targeting PTSD and SUD concurrently.^75^,^76^ This integrated focus is particularly relevant to women who present to SUD treatment and often have elevated rates of trauma history and PTSD.^12^

Recently, integrated models of treatment for PTSD and SUD have been developed and tested with mixed results. For instance, Najavits developed Seeking Safety (SS), a CBT-based treatment model that aims to reduce co-occurring PTSD and SUD by enhancing coping skills.^77^ SS emphasizes themes of establishing safety, taking back power, being honest, setting boundaries, practicing compassion, healing from anger, grounding, creating meaning, and increasing self-care. Hien and colleagues tested the efficacy of SS and another active treatment condition Relapse Prevention against a treatment-as-usual control condition.^78^ Women in SS and relapse prevention had comparable posttreatment reductions in both PTSD and SUD symptoms, and both treatments were superior to the control condition. Likewise, a study conducted through the National Institute on Drug Abuse Clinical Trials Network found no differences in PTSD or SUD outcomes between an abbreviated version of SS and a health education control condition, both delivered as adjuncts to standard SUD treatment.^79^

Morrissey and colleagues studied another integrated treatment approach for women with SUD.^80^ The researchers used a quasi-experimental design to examine a large cohort treated across nine sites. Participants were mostly of low socioeconomic status and had serious mental and/or physical health problems as well as an interpersonal trauma history. The integrated treatment was associated with lower substance use and improved general mental health but not with reduced PTSD symptoms. Overall, it remains unclear whether integrated treatments for PTSD and AUD/SUD in women are superior to stand-alone SUD treatments. Widespread methodological limitations in the current literature warrant continued investigation of integrated treatments, including outcomes that may be specific to women with AUD.^75^,^76^

#### Mood disorders

Another promising area of treatment development for women is integrated behavioral therapy for SUD and depression. Treating depression and AUD concurrently may be important because negative affect is a particularly salient trigger for drinking among women. In turn, regular heavy drinking may inhibit recovery from mood disorders. Further, more women than men with AUD have a co-occurring mood disorder, and there is an elevated suicide risk among women with AUD.^6^ However, research on integrated AUD and mood disorder treatments for women is limited. For example, in a pilot study, researchers tested 8 sessions of interpersonal psychotherapy as an adjunct to outpatient AUD treatment for 14 women with co-occurring AUD and major depression.^81^ The study found that women were highly engaged and satisfied with the adjunct treatment and reported follow-up reductions in drinking, depressive symptoms, and interpersonal problems. A study of men and women with depressive symptoms and hazardous drinking compared the effects of integrated alcohol-depression treatment, alcohol-only treatment, and depression-only treatment.^82^ The integrated treatment generally produced the best alcohol and depression outcomes for both women and men. In the nonintegrated treatments, women’s drinking and depressive symptoms improved more in the depression-only treatment, whereas men improved more in the alcohol-only treatment. These findings highlight the unique benefit of treating depression among women with co-occurring AUD and suggest the need for more RCTs targeting this co-occurrence in women.

Given that drinking and antidepressant use are generally contraindicated adds to the significance of concurrent treatment of AUD and depression to maximize the effectiveness of psychotropic medications.^6^ One RCT tested the effect of citalopram plus naltrexone and clinical case management for men and women with AUD and depression.^83^ Compared to placebo, citalopram did not produce greater improvements in drinking or mood with one exception: women (but not men) on citalopram had a higher percentage of abstinent days. These findings point to the potential for tailoring antidepressant treatment to maximize treatment benefits for women with co-occurring AUD and depression.

#### Borderline personality disorder

Research has demonstrated elevated rates (i.e., of approximately 18%) of borderline personality disorder (BPD) in women seeking treatment for AUD.^84^ Dialectical behavior therapy (DBT) is an empirically supported treatment for BPD that has been successfully adapted for co-occurring SUD.^85^ A systematic review found that DBT has shown positive potential for the treatment of women with co-occurring SUD and BPD,^86^ leading to reductions in substance use, suicidal/self-injurious behaviors, treatment attrition, and social functioning problems. No studies that tested DBT specifically with women who have co-occurring AUD and BPD have been found.

### Mechanisms of Change: How Change Occurs

The goal of understanding moderators and mechanisms of change in treatment is to identify how patient characteristics interact with treatments, identify variables key to successful change, and then develop or modify treatments to target those variables more efficiently in treatment. Currently, there are relatively limited data on moderators and mechanisms of change in alcohol use during and after AUD treatment for women. Moderators are defined as “specification variables” that impact the association between two other variables,^87^ for instance, the effect of baseline major depressive disorder on treatment outcome of female-specific versus gender-neutral treatment for AUD. A mediator is an “intervening variable” that “transmits the effect of the independent variable on the dependent variable”;^87^ for instance, cognitive behavioral treatment of AUD has its effect on drinking outcome in part by increased use of effective coping skills among clients.

Research on moderators of outcome has elucidated the need for heterogeneity in samples and helped to refine female-specific treatments.^87^ For example, findings that anxiety pretreatment and depression pre- and posttreatment predicted poorer drinking outcomes for women^88^ suggest the value of including interventions to alleviate depression and anxiety in female-specific AUD treatment. Recent and more sophisticated research has studied the interaction of moderators and mediators of treatment response. For instance, Holzhauer and colleagues combined a moderator analysis with testing the intensity and timing of reductions in drinking after specific outpatient treatment sessions that targeted depression and anxiety in female-specific AUD treatment.^89^ Three moderators assessed at baseline—depression, anxiety, and self-efficacy to remain abstinent in negative affect situations—predicted sudden gains (i.e., a steep decrease in drinking) after Session 5 or 6, which included interventions to attenuate negative affect. The results suggest that women who enter treatment struggling with negative affect may respond well to very specific, targeted interventions for those problems.

Hallgren and colleagues examined three hypothesized mechanisms of change—abstinence self-efficacy, coping skills, and therapeutic alliance—in outpatient AUD treatment for women.^90^ These authors used daily data from the individual versus group female-specific parent study^60^ and sophisticated longitudinal statistical modeling to quantify rates of change around initiation of abstinence for each participant in outpatient FS-CBT. They also tested time-linked change in mediators before each of the 12 therapy sessions. Data on daily drinking and craving were available for the baseline, in-treatment, and 12-month follow-up periods. Results focused on two subgroups of women: those who had initiated abstinence before treatment and those who initiated abstinence during treatment. Those who initiated abstinence during treatment showed marked improvements in two key hypothesized mechanisms of change (abstinence self-efficacy and coping skills) during the week that they initiated abstinence. Women who were abstinent at the start of treatment maintained higher abstinence self-efficacy and coping skills throughout treatment. Previously, Hallgren and colleagues had found that daily-rated alcohol craving (a different mediator) decreased in relation to initiation of abstinence in men and women in outpatient CBT for AUD.^91^

Using Network Analysis, a novel statistical approach that uses multilevel vector autoregression estimation for multiple time series data to simultaneously examine change among several hypothesized mechanisms of change, Holzhauer and colleagues compared pathways to drinking reduction among women in gender-neutral versus FS-CBT.^59^,^92^ Across treatments, women changed their drinking via increased coping skills, abstinence self-efficacy, and increased autonomy. For women in FS-CBT, change in drinking also occurred through decreases in sociotropy and increases in social support for abstinence. Surprisingly, change in depression was linked to better drinking outcomes for women in gender-neutral CBT.

Going forward, continuing moderated mediation studies that examine the response of gender-specific moderators of response to medications or behavioral interventions for AUD, and the mechanisms by which these treatments operate for specific subpopulations, will help guide the development of personalized medicine for addiction.^30^ A moderated mediation approach can facilitate examination of individual differences and sample heterogeneity that are linked to drinking outcomes and help to identify gender differences in pathways to successful treatment outcomes.

## CONCLUSIONS AND RECOMMENDATIONS

Since the National Institutes of Health mandate in 1994 that biomedical research include female participants in clinical research,^93^ a substantive body of literature emerged describing the unique aspects of AUD among women, which led to an accelerated development of treatments targeting women’s unique clinical presentation. In 2006, the National Institute on Alcohol Abuse and Alcoholism (NIAAA) identified women as an understudied population in treatment research and prioritized research to better understand the mechanisms by which treatments for AUD effect change in drinking.^94^,^95^ Findings that drinking outcomes of female-specific and gender-neutral treatments may be similar does not mean that the development of female-specific treatments should not be pursued. First, there is evidence that mechanisms of women’s response to treatment (i.e., pathways to change) may differ from that of men, and identification of these gender-specific pathways can guide the development of efficient, gender-differentiated active ingredients in treatment. Second, there may be greater benefits of women-specific (vs. gender-neutral) treatment for secondary outcomes, such as psychosocial well-being, psychiatric health, pregnancy outcomes, and HIV risk reduction. Third, further study is needed on whether the availability of women-specific and women-only treatments enhances treatment access and engagement for women with AUD.

Gaps in knowledge remain; however, increasingly sophisticated research approaches are available to continue to tackle the questions of how and which treatments work best for whom. The contemporary focus on personalized medicine^96^,^97^ extends to women with AUD; the end goal is not only to provide an array of specialized treatment options specifically tailored to enhance women’s treatment access and engagement but also to provide science-based treatment elements and options uniquely matched to various common clinical presentations among women with AUD.

A critical problem to resolve is treatment access and utilization. Only 15% of women with lifetime AUD ever seek treatment for it, and women experience multiple individual-based barriers to accessing treatment. In addition, systemic barriers to AUD treatment for women need attention, as a minority of substance use treatment services in the United States offer gender-segregated or female-specific programming. Extant literature suggests that women may prefer gender-segregated treatment for AUD but also suggests this treatment offers no added benefit in the absence of female-specific programming content. Thus, widespread availability of female-only treatment settings that include evidence-based female-specific interventions and content is likely to increase treatment utilization and enhance outcomes for women with AUD. In order to populate female-only treatment settings with female-specific programming, we need to develop an array of evidence-based options. A number of RCTs have yielded newly available, evidence-based female-specific treatment protocols for AUD and SUD treatment that are at least equivalent in positive outcomes to evidence-based control treatments.^59^,^60^,^62^,^70^,^74^,^79^ Outcomes for secondary (non-AUD) patient problems, such as depression and anxiety,^59^,^60^ trauma symptoms,^69^ cardiovascular function,^98^ health behaviors, drug use, and quality of life^99^,^100^ from these female-specific treatments also have been positive. NIAAA’s focus on implementation studies in conjunction with the study of mechanisms of change^101^ should accelerate testing the incorporation of female-specific interventions into community settings—not just addiction specialty clinics but also primary care and general mental health settings. These interventions should ultimately lead to algorithms for optimal personalization of treatment components to individuals’ clinical presentation. In the meantime, since most women currently receive treatment in gender-neutral settings, it is important to address women’s specific needs even in the context of mixed-gender, gender-neutral^102^ clinical programming. Research to address unresolved gaps in the knowledge base is needed. For example, does the availability of female-specific programming, whether in female-segregated or mixed-gender settings, increase AUD treatment utilization by women? In addition, there is a dearth of rigorous RCTs comparing female-only versus mixed-gender treatment formats that contain female-specific programming to test differential treatment engagement and positive outcomes.

Notable areas of additional needed research on women and AUD treatment follow.

### Prevention

Women who enter treatment for AUD present with greater addiction and more severe psychosocial issues than men. Secondary prevention research has focused on engaging women in treatment as well as on providing alcohol psychoeducation earlier in women’s problem drinking careers, which may help arrest the telescoped trajectory to AUD and SUD and the corresponding psychosocial decline.

### Setting

Women are more likely to self-identify as having an alcohol problem and enter AUD treatment through a medical or mental health portal than a substance use specialty clinic. For instance, women may obtain AUD treatment in the course of seeking treatment for a co-occurring psychiatric disorder, such as PTSD or depression, in a general mental health setting.^19^,^20^ Also, brief interventions in primary care settings have been found to be promising in reducing drinking among less complex cases of women with low co-occurrence,^103^ but no studies have examined the co-location of more intensive outpatient female-specific AUD treatments in primary care or women’s medical clinic settings.

### Treatment Silos

Increasing rates of drug use among women point to a need for integrated AUD and SUD female-specific treatments. Although some evidence-based treatments are available,^103^ the net can be cast even wider to include a range of health behaviors such as nutrition, sleep, exercise, smoking cessation, and use of benzodiazepines. Framing AUD treatment for women in the context of a general health and wellness approach that addresses other health behaviors may increase appeal, reduce stigma, and enhance utilization.

### Digital Delivery Platforms

Testing telehealth platforms for individual and group AUD treatments may help reduce barriers to use among women. Likewise, testing ancillary smartphone applications that link women to in vivo coping skills training and social network support could enhance outcomes of existing in-person programs or serve as stand-alone aids for women who face insurmountable treatment entry barriers.

### Female-Specific, Coping-Skills-Based, Peer Support Groups

Female-specific, coping-skills-based, peer support groups are not widely available. The evidence base for women’s Alcoholics Anonymous meetings needs to be established. In addition, the recent positive development of a recovery coach industry may help with in vivo social support especially for women, but research is necessary to establish an evidence base.

### Medications

Research on medications for women with AUD as one treatment element should continue. A precision medicine approach testing gender, genetic profiles, and specific medications is an important avenue to pursue.

### Mechanisms of Change Research

Research on mechanisms of change is crucial to untangle whether similar drinking outcomes of women and men with AUD are achieved via gender-specific pathways to change and to identify active ingredients and mediators of treatment change best suited for women with only AUD and for women with specific types of co-occurring disorders. New methodologies in statistics, neuroscience, and research design are helping to clarify these questions; however, additional research is needed to streamline and personalize optimally efficient treatment components for every woman seeking care for AUD.

## References

[1] National Institute of Alcohol Abuse and Alcoholism (NIAAA), National Institutes of Health (2018). Alcohol Facts and Statistics.

[2] McCaul ME, Roach D, Hasin DS (2019). Alcohol and women: A brief overview. Alcohol Clin Exp Res.

[3] Witbrodt J, Mulia N, Zemore SE (2014). Racial/ethnic disparities in alcohol-related problems: Differences by gender and level of heavy drinking. Alcohol Clin Exp Res.

[4] McCabe SE, West BT, Hughes TL (2013). Sexual orientation and substance abuse treatment utilization in the United States: Results from a national survey. J Subst Abuse Treat.

[5] Alvanzo AAH, Storr CL, Mojtabai R (2014). Gender and race/ethnicity differences for initiation of alcohol-related service use among persons with alcohol dependence. Drug Alcohol Depend.

[6] Epstein EE, Menges D, McCrady BS, Epstein EE (2013). Women and addiction. Addictions: A Comprehensive Guidebook.

[7] Substance Abuse and Mental Health Services Administration (SAMHSA), Center for Substance Abuse Treatment (2013). Substance Abuse Treatment: Addressing the Specific Needs of Women. A Treat Improvement Protocol TIP.

[8] Oliva EM, Gregor A, Rogers J (2012). Correlates of specialty substance use disorder treatment among female patients in the Veterans Health Administration. J Soc Work Pract Addict.

[9] Khan S, Okuda M, Hasin DS (2013). Gender differences in lifetime alcohol dependence: Results from the National Epidemiologic Survey on Alcohol and Related Conditions. Alcohol Clin Exp Res.

[10] Kalpakci A, Sofuoglu M, Petrakis I (2019). Gender differences among Veterans with alcohol use disorder nationally in the Veterans Health Administration. J Addict Dis.

[11] Pirard S, Sharon E, Kang SK (2005). Prevalence of physical and sexual abuse among substance abuse patients and impact on treatment outcomes. Drug Alcohol Depend.

[12] Hien DA, Litt L, Cohen LC (2009). Integrating Trauma Services for Women in Addictions Treatment.

[13] Wu L-T, Ringwalt CL (2004). Alcohol dependence and use of treatment services among women in the community. Am J Psychiatry.

[14] Lale R, Sklar M, Wooldridge J (2014). Gender congruence moderates beliefs about the causes of alcohol dependence and major depression. Int J Ment Health Addict.

[15] Verissimo ADO, Grella CE (2017). Influence of gender and race/ethnicity on perceived barriers to help-seeking for alcohol or drug problems. J Subst Abuse Treat.

[16] Rodriguez LM, Neighbors C, Knee CR (2014). Problematic alcohol use and marital distress: An interdependence theory perspective. Addict Res Theory.

[17] Tuchman E (2010). Women and addiction: The importance of gender issues in substance abuse research. J Addict Dis.

[18] Gilbert PA, Pro G, Zemore SE (2019). Gender differences in use of alcohol treatment services and reasons for nonuse in a national sample. Alcohol Clin Exp Res.

[19] Edlund MJ, Booth BM, Han X (2012). Who seeks care where? Utilization of mental health and substance use disorder treatment in two national samples of individuals with alcohol use disorders. J Stud Alcohol Drugs.

[20] Manuel JI, Stebbins MB, Wu E (2018). Gender differences in perceived unmet treatment needs among persons with and without co-occurring disorders. J Behav Health Serv Res.

[21] Grella CE, Brady KT, Back SE, Greenfield SF (2009). Treatment seeking and utilization among women with substance use disorders. Women and Addiction: A Comprehensive Handbook.

[22] Green CA (2006). Gender and use of substance abuse treatment services. Alcohol Res Health.

[23] Greenfield SF, Brooks AJ, Gordon SM (2007). Substance abuse treatment entry, retention, and outcome in women: A review of the literature. Drug Alcohol Depend.

[24] Green P, Watts D, Poole S (2004). Why patients sign out against medical advice (AMA): Factors motivating patients to sign out AMA. Am J Drug Alcohol Abuse.

[25] Bravo F, Gual A, Lligoña A (2013). Gender differences in the long-term outcome of alcohol dependence treatments: An analysis of twenty-year prospective follow up. Drug Alcohol Rev.

[26] Bornstein K, Longinaker N, Bryant-Genevier M (2015). Sex differences in substance abuse treatment adherence in the United States. Addict Disord Their Treat.

[27] Rash CJ, Petry NM (2015). Contingency management treatments are equally efficacious for both sexes in intensive outpatient settings. Exp Clin Psychopharmacol.

[28] Litt MD, Kadden RM, Tennen H (2015). Network Support treatment for alcohol dependence: Gender differences in treatment mechanisms and outcomes. Addict Behav.

[29] Unger A, Jung E, Winklbaur B (2014). Gender issues in the pharmacotherapy of opioid-addicted women: Buprenorphine. Women, Children, and Addiction.

[30] Agabio R, Pani PP, Preti A (2016). Efficacy of medications approved for the treatment of alcohol dependence and alcohol withdrawal syndrome in female patients: A descriptive review. Eur Addict Res.

[31] Canidate SS, Carnaby GD, Cook CL (2017). A systematic review of naltrexone for attenuating alcohol consumption in women with alcohol use disorders. Alcohol Clin Exp Res.

[32] Ashley OS, Marsden ME, Brady TM (2003). Effectiveness of substance abuse treatment programming for women: A review. Am J Drug Alcohol Abuse.

[33] Sword W, Jack S, Niccols A (2009). Integrated programs for women with substance use issues and their children: A qualitative meta-synthesis of processes and outcomes. Harm Reduct J.

[34] SAMHSA (2019). National Survey of Substance Abuse Treatment Services (N-SSATS): 2018. Data on Substance Abuse Treatment Facilities.

[35] Timko C, Hoggatt KJ, Wu FM (2017). Substance use disorder treatment services for women in the Veterans Health Administration. Womens Health Issues.

[36] Smith WB, Weisner C (2000). Women and alcohol problems: A critical analysis of the literature and unanswered questions. Alcohol Clin Exp Res.

[37] Greenfield SF, Grella CE (2009). What is “women-focused” treatment for substance use disorders?. Psychiatr Serv.

[38] Greenfield SF, Pirard S, Brady KT, Back SE, Greenfield SF (2009). Gender-specific treatment for women with substance use disorders. Women and Addiction: A Comprehensive Handbook.

[39] Neale J, Tompkins CNE, Marshall AD (2018). Do women with complex alcohol and other drug use histories want women-only residential treatment?. Addiction.

[40] Bride BE (2001). Single-gender treatment of substance abuse: Effect on treatment retention and completion. Soc Work Res.

[41] Giannitrapani KF, Huynh AK, Schweizer CA (2018). Patient-centered substance use disorder treatment for women Veterans. J Mil Veteran Fam Health.

[42] Dahlgren L, Willander A (1989). Are special treatment facilities for female alcoholics needed? A controlled 2-year follow-up study from a specialized female unit (EWA) versus a mixed male/female treatment facility. Alcohol Clin Exp Res.

[43] Gjestad R, Franck J, Lindberg S (2011). Early treatment for women with alcohol addiction (EWA) reduces mortality: A randomized controlled trial with long-term register follow-up. Alcohol Alcohol.

[44] Grella CE (1999). Women in residential drug treatment: Differences by program type and pregnancy. J Health Care Poor Underserved.

[45] Grella CE, Joshi V, Hser YI (2000). Program variation in treatment outcomes among women in residential drug treatment. Eval Rev.

[46] Claus RE, Orwin RG, Kissin W (2007). Does gender-specific substance abuse treatment for women promote continuity of care?. J Subst Abuse Treat.

[47] Niv N, Hser Y-I (2007). Women-only and mixed-gender drug abuse treatment programs: Service needs, utilization and outcomes. Drug Alcohol Depend.

[48] Prendergast ML, Messina NP, Hall EA (2011). The relative effectiveness of women-only and mixed-gender treatment for substance-abusing women. J Subst Abuse Treat.

[49] Kaskutas LA, Zhang L, French MT (2005). Women’s programs versus mixed-gender day treatment: Results from a randomized study. Addiction.

[50] McCrady BS, Epstein EE, Cook S (2009). A randomized trial of individual and couple behavioral alcohol treatment for women. J Consult Clin Psychol.

[51] Schumm JA, O’Farrell TJ, Kahler CW (2014). A randomized clinical trial of behavioral couples therapy versus individually based treatment for women with alcohol dependence. J Consult Clin Psychol.

[52] O’Farrell TJ, Schumm JA, Murphy MM (2017). A randomized clinical trial of behavioral couples therapy versus individually-based treatment for drug-abusing women. J Consult Clin Psychol.

[53] McCrady BS, Epstein EE, Cook S (2011). What do women want? Alcohol treatment choices, treatment entry and retention. Psychol Addict Behav.

[54] Upshur C, Weinreb L, Bharel M (2015). A randomized control trial of a chronic care intervention for homeless women with alcohol use problems. J Subst Abuse Treat.

[55] Polcin DL, Nayak MB, Korcha R (2019). Heavy drinking among women receiving intensive motivational interviewing: 6-month outcomes. J Psychoactive Drugs.

[56] Connors GJ, Walitzer KS (2001). Reducing alcohol consumption among heavily drinking women: Evaluating the contributions of life-skills training and booster sessions. J Consult Clin Psychol.

[57] Walitzer KS, Connors GJ (2007). Thirty-month follow-up of drinking moderation training for women: A randomized clinical trial. J Consult Clin Psychol.

[58] Epstein EE, McCrady BS (2009). Overcoming Alcohol Use Problems: A Cognitive-Behavioral Treatment Program.

[59] Epstein EE, McCrady BS, Hallgren KA (2018). A randomized trial of female-specific cognitive behavior therapy for alcohol dependent women. Psychol Addict Behav.

[60] Epstein EE, McCrady BS, Hallgren KA (2018). Individual versus group female-specific cognitive behavior therapy for alcohol use disorder. J Subst Abuse Treat.

[61] Olmstead TA, Graff FS, Ames-Sikora A (2019). Cost-effectiveness of individual versus group female-specific cognitive behavioral therapy for alcohol use disorder. J Subst Abuse Treat.

[62] Greenfield SF, Trucco EM, McHugh RK (2007). The Women’s Recovery Group study: A Stage I trial of women-focused group therapy for substance use disorders versus mixed-gender group drug counseling. Drug Alcohol Depend.

[63] Greenfield SF (2016). Treating Women with Substance Use Disorders: The Women’s Recovery Group Manual.

[64] McHugh RK, Greenfield SF (2010). Psychiatric symptom improvement in women following group substance abuse treatment: Results from the Women’s Recovery Group study. J Cogn Psychother.

[65] Greenfield SF, Sugarman DE, Freid CM (2014). Group therapy for women with substance use disorders: Results from the Women’s Recovery Group Study. Drug Alcohol Depend.

[66] Greenfield SF, Kuper LE, Cummings AM (2013). Group process in the single-gender Women’s Recovery Group compared with mixed-gender Group Drug Counseling. J Groups Addict Recover.

[67] Sugarman DE, Wigderson SB, Iles BR (2016). Measuring affiliation in group therapy for substance use disorders in the Women’s Recovery Group study: Does it matter whether the group is all-women or mixed-gender?. Am J Addict.

[68] Valeri L, Sugarman DE, Reilly ME (2018). Group therapy for women with substance use disorders: In-session affiliation predicts women’s substance use treatment outcomes. J Subst Abuse Treat.

[69] Sugarman DE, Meyer LE, Reilly ME (2019). Feasibility and acceptability of a web-based, gender-specific intervention for women with substance use disorders. J Women’s Health.

[70] Najavits LM, Enggasser J, Brief D (2018). A randomized controlled trial of a gender-focused addiction model versus 12-step facilitation for women veterans. Am J Addict.

[71] Covington SS (2008). Helping Women Recover: A Program for Treating Addiction.

[72] Covington SS (2003). Beyond Trauma: A Healing Journey for Women.

[73] Messina N, Grella CE, Cartier J (2010). A randomized experimental study of gender-responsive substance abuse treatment for women in prison. J Subst Abuse Treat.

[74] Saxena P, Messina NP, Grella CE (2014). Who benefits from gender-responsive treatment? Accounting for abuse history on longitudinal outcomes for women in prison. Crim Justice Behav.

[75] Flanagan JC, Korte KJ, Killeen TK (2016). Concurrent treatment of substance use and PTSD. Curr Psychiatry Rep.

[76] Simpson TL, Lehavot K, Petrakis IL (2017). No wrong doors: Findings from a critical review of behavioral randomized clinical trials for individuals with co-occurring alcohol/drug problems and posttraumatic stress disorder. Alcohol Clin Exp Res.

[77] Najavits L (2002). Seeking Safety: A Treatment Manual for PTSD and Substance Abuse.

[78] Hien DA, Cohen LR, Miele GM (2004). Promising treatments for women with comorbid PTSD and substance use disorders. Am J Psychiatry.

[79] Hien DA, Wells EA, Jiang H (2009). Multisite randomized trial of behavioral interventions for women with co-occurring PTSD and substance use disorders. J Consult Clin Psychol.

[80] Morrissey JP, Ellis AR, Gatz M (2005). Outcomes for women with co-occurring disorders and trauma: Program and person-level effects. J Subst Abuse Treat.

[81] Gamble SA, Talbot NL, Cashman-Brown SM (2013). A pilot study of interpersonal psychotherapy for alcohol-dependent women with co-occurring major depression. Subst Abus.

[82] Baker AL, Kavanagh DJ, Kay-Lambkin FJ (2010). Randomized controlled trial of cognitive-behavioural therapy for coexisting depression and alcohol problems: Short-term outcome. Addiction.

[83] Adamson SJ, Sellman JD, Foulds JA (2015). A randomized trial of combined citalopram and naltrexone for nonabstinent outpatients with co-occurring alcohol dependence and major depression. J Clin Psychopharmacol.

[84] Rosenthal RN, McCrady BS, Epstein EE (2013). Treatment of persons with substance use disorder and co-occurring other mental disorders. Addictions: A Comprehensive Guidebook.

[85] Dimeff LA, Linehan MM (2008). Dialectical behavior therapy for substance abusers. Addict Sci Clin Pract.

[86] Lee NK, Cameron J, Jenner L (2015). A systematic review of interventions for co-occurring substance use and borderline personality disorders. Drug Alcohol Rev.

[87] Longabaugh R, Magill M, Morgenstern J, McCrady BS, Epstein EE (2013). Mechanisms of behavior change in treatment for alcohol and other drug use disorders. Addictions: A Comprehensive Guidebook.

[88] Haver B, Gjestad R (2005). Phobic anxiety and depression as predictor variables for treatment outcome. A LISREL analysis on treated female alcoholics. Nord J Psychiatry.

[89] Holzhauer CG, Epstein EE, Hayaki J (2017). Moderators of sudden gains after sessions addressing emotion regulation among women in treatment for alcohol use. J Subst Abuse Treat.

[90] Hallgren KA, Epstein EE, McCrady BS (2019). Changes in hypothesized mechanisms of change before and after initiating abstinence in cognitive-behavioral therapy for women with alcohol use disorder. Behav Ther.

[91] Hallgren KA, McCrady BS, Epstein EE (2016). Trajectories of drinking urges and the initiation of abstinence during cognitive–behavioral alcohol treatment. Addiction.

[92] Holzhauer CG, Hildebrandt T, Epstein EE (2020). Mechanisms of change in female-specific and gender-neutral cognitive behavioral therapy for women with alcohol use disorder. J Consult Clin Psychol.

[93] Food and Drug Administration, National Institutes of Health, Department of Health and Human Services (1994). NIH Guidelines on the Inclusion of Women and Minorities as Subjects in Clinical Research.

[94] NIAAA (2006). Mechanisms of Behavior Change Research Initiative: Strategic Research Plan.

[95] NIAAA (2006). Mechanisms of Behavior Change in the Treatment of Alcohol Use Disorders (R21).

[96] Precision Medicine Initiative Working Group (2015). The Precision Medicine Initiative Cohort Program – Building a Research Foundation for 21st Century Medicine.

[97] Willenbring ML (2010). The past and future of research on treatment of alcohol dependence. Alcohol Res Health.

[98] Buckman JF, Vaschillo B, Vaschillo EG (2019). Improvement in women’s cardiovascular functioning during treatment for alcohol use disorder. Psychol Addict Behav.

[99] Bold KW, Epstein EE, McCrady BS (2017). Baseline health status and quality of life after alcohol treatment for women with alcohol dependence. Addict Behav.

[100] Epstein EE, McCrady BS, Cook S (2015). Non-ETOH drug use among women in outpatient treatment for alcohol dependence. Drug Alcohol Depend.

[101] NIAAA (2019). Notice of Special Interest on Development and Dissemination of Behavioral Treatments for AUD.

[102] SAMHSA (2016). Guidance Document for Supporting Women in Co-Ed Settings.

[103] Cucciare MA, Simpson T, Hoggatt KJ (2013). Substance use among women veterans: Epidemiology to evidence-based treatment. J Addict Dis.

